# Increasing cleavage specificity and activity of restriction endonuclease KpnI

**DOI:** 10.1093/nar/gkt734

**Published:** 2013-08-19

**Authors:** Kommireddy Vasu, Easa Nagamalleswari, Mai Zahran, Petra Imhof, Shuang-yong Xu, Zhenyu Zhu, Siu-Hong Chan, Valakunja Nagaraja

**Affiliations:** ^1^Department of Microbiology and Cell Biology, Indian Institute of Science, Bangalore 560 012, India, ^2^Department of Chemistry, New York University, NY, USA, ^3^Department of Physics, Free University Berlin, Arnimallee 14, 14195 Berlin, Germany, ^4^New England Biolabs, Inc., 240 County Road, Ipswich, MA 01938, USA and ^5^Jawaharlal Nehru Centre for Advanced Scientific Research, Bangalore 560 064, India

## Abstract

Restriction enzyme KpnI is a HNH superfamily endonuclease requiring divalent metal ions for DNA cleavage but not for binding. The active site of KpnI can accommodate metal ions of different atomic radii for DNA cleavage. Although Mg^2+^ ion higher than 500 μM mediates promiscuous activity, Ca^2+^ suppresses the promiscuity and induces high cleavage fidelity. Here, we report that a conservative mutation of the metal-coordinating residue D148 to Glu results in the elimination of the Ca^2+^-mediated cleavage but imparting high cleavage fidelity with Mg^2+^. High cleavage fidelity of the mutant D148E is achieved through better discrimination of the target site at the binding and cleavage steps. Biochemical experiments and molecular dynamics simulations suggest that the mutation inhibits Ca^2+^-mediated cleavage activity by altering the geometry of the Ca^2+^-bound HNH active site. Although the D148E mutant reduces the specific activity of the enzyme, we identified a suppressor mutation that increases the turnover rate to restore the specific activity of the high fidelity mutant to the wild-type level. Our results show that active site plasticity in coordinating different metal ions is related to KpnI promiscuous activity, and tinkering the metal ion coordination is a plausible way to reduce promiscuous activity of metalloenzymes.

## INTRODUCTION

Restriction endonucleases (REases) exhibit high sequence specificity in substrate binding and use versatile DNA cleavage mechanisms and thus are excellent model systems for understanding DNA recognition and phosphodiester bond hydrolysis. Many REases studied so far belong to the PD-(D/E)XK superfamily of nucleases (1). However, based on structural, biochemical and sequence analyses, it was shown that REases use not only the PD-(D/E)XK fold but also unrelated catalytic domains found in other classes of nucleases, namely, phospholipase D (PLD), GIY-YIG, half pipe and HNH families (1–7). Of these five evolutionarily distinct active site motifs, the HNH superfamily of REases forms the second largest group and is characterized by the presence of the ββα-Me finger fold (2,7). Nucleases of this superfamily are present in all three kingdoms of life and exhibit broad specificity ranging from sequence- or structure-specific to completely non-specific to sequence or structure (8,9).

Studies carried out to date on HNH nucleases imply a single metal ion-mediated catalysis (9,10). For hydrolysis, a conserved His in the active site acts as a general base to activate the nucleophilic water molecule, which then attacks the scissile phosphodiester bond. The role of the coordinated metal ion serves two purposes—stabilization of the transition state and coordination of a water molecule to protonate the leaving group (11). It has been suggested that this single-metal ion mechanism imposes a less stringent requirement on the spatial coordination of the metal ion than in the two-metal ion mechanism used by many PD-(D/E)XK REases and therefore allows HNH nucleases to exhibit a broader metal ion co-factor requirement (8,12,13). In general, HNH enzymes exhibit preference for metal ions of a particular type, i.e. either alkaline earth or transition group (10,11,14–18). KpnI, the first REase identified as a member of HNH superfamily, however, can use a battery of divalent metal ion co-factors belonging to both alkaline earth and transition groups for DNA cleavage (19).

Many REases show relatively low degree of promiscuous activity under standard reaction conditions in the presence of Mg^2+^ but exhibit significant star activity under altered conditions, namely, low ionic strength, elevated pH or when Mg^2+^ is substituted with Mn^2+^ (20). KpnI, however, exhibits significant promiscuous activity in the presence of Mg^2+^ (21). Interestingly, the promiscuous cleavage is suppressed when the metal ion is replaced by Ca^2+^: in the presence of Ca^2+^ (ionic radius = 1.14 Å), the enzyme is specific even at high concentrations of the enzyme or the metal ion, whereas in the presence of Mg^2+^ (ionic radius = 0.86 Å), KpnI exhibits promiscuous activity even at low enzyme concentrations (21). Promiscuous activity has been proposed to offer an adaptive fitness to the host bacteria (22) and a gateway to the evolution of new enzyme activities (23,24). The dramatic effect of metal ion cofactor on the KpnI activity is intriguing and suggests that altering the active site interactions with the metal ion could affect the enzyme’s specificity. In our previous studies, we reported that the mutation of several residues in KpnI altered its metal ion binding properties and enhanced cleavage fidelity (25–27). We describe here the generation of a KpnI variant with an extremely low promiscuity by introducing a conservative mutation of a metal-coordinating residue. The mutant also becomes inactive with Ca^2+^. Although the high fidelity mutant showed a lower specific activity, a suppressor mutation that enhances the specific activity of the enzyme has been identified. Our results show that altering the metal ion-binding properties of KpnI, and possibly other HNH REases, can alter its substrate specificity and reinforce the notion that the bound metal ion is correlated to the promiscuous activity of the enzyme.

## MATERIALS AND METHODS

### Enzymes, chemicals and DNA

KpnI and its mutants were purified from *Escherichia coli* NEB Express (New England Biolabs) expressing the KpnI or mutant proteins from a plasmid under the control of a *lac* promoter and M.KpnI from a compatible plasmid under the control of a *tet* promoter (unpublished results). About 9 g of wet cell paste were resuspended in 100 ml of Buffer A [20 mM Tris–HCl (pH 8.0) 50 mM NaCl, 1 mM ethylenediaminetetraacetic acid (EDTA), 10 mM phenylmethylsulfonyl fluoride] and sonicated. After centrifugation at 15 000 rpm for 30 min at 4°C in a JA-17 rotor (Beckman Coulter), the supernatant was loaded onto a Heparin HyperDM column (20 ml; Pall). Protein was eluted by a linear gradient of 50–1000 mM NaCl in Buffer A over 20 column volumes at a flow rate of 2 ml/min. Peak fractions were pooled and diluted 4-fold into 20 mM potassium phosphate buffer (pH 7.0), 50 mM NaCl, 1 mM EDTA (Buffer B) and then loaded onto a ceramic hydroxylapatite column (7 ml; Bio-Rad). Protein was eluted using a linear gradient of 50–500 mM potassium phosphate in Buffer B over 40 column volumes at a flow rate of 2 ml/min. Peak fractions were pooled, concentrated and buffer-exchanged to a buffer containing 40 mM Tris–HCl (pH 8.0), 500 mM NaCl, 1 mM EDTA using Vivaspin 15 (Sartorius Stedim Biotech). After addition of equal volume of 100% glycerol, the concentrated proteins were stored at −20°C. Protein concentration was determined by measuring its absorbance at 280 nm and using a molar extinction coefficient of 45 000 M^−1 ^cm^−1^. The purity of the proteins was >95% as judged by SDS-PAGE followed by Coomassie Blue staining.

The enzymes were diluted in a buffer containing 20 mM Tris–HCl (pH 8.0), 25 mM NaCl and 5 mM 2-mercaptoethanol for all the experiments. Oligonucleotides (Sigma) were gel purified and 5′-end labeled using T4 polynucleotide kinase (New England Biolabs) and [γ-^32^P] ATP (6000 Ci/mmol; PerkinElmer). The labeled oligonucleotides were purified using G-25 Sephadex spin column chromatography.

### Mutagenesis and screening for higher cleavage activity

Site-directed mutagenesis was carried out essentially as described using a modified inverse PCR scheme (28). To find a suppressor mutation that can reverse the reduced activity of the high fidelity mutant D148E, selected residues were mutated to Ala using Vent DNA polymerase (New England Biolabs). *Escherichia coli* NEB Express carrying a plasmid expressing M.KpnI was used as competent cells for transformation by the inverse PCR products. Mutants with higher specific activity were screened for as follows: 1 ml of overnight culture of NEB Express carrying the mutants were harvested, sonicated and centrifuged. Ten-fold dilutions of the supernatant were assayed using plasmid pXba (a pUC19 derivative carrying a XbaI fragment of adenovirus DNA) as substrate in NEBuffer 4 (New England Biolabs) at 37°C for 1 h, followed by 0.8% agarose gel electrophoresis. Mutation(s) were verified by DNA sequencing of the entire *kpnIR* gene.

### Intrinsic fluorescence

Fluorescence emission spectra were recorded using Jobin-Yvon fluorometer FluoroMax 3 (HORIBA, Jobin-Yvon/Spex Division, Longjumeau, France), thermostated at 25°C. The change in the tryptophan fluorescence was measured at excitation and emission wavelengths of 295 and 340 nm, respectively, with a 5 nm slit width. For all the studies, proteins were dialyzed against EDTA to remove the intrinsically bound metal cofactor, and EDTA was removed by dialysis against 10 mM Tris–HCl (pH 7.4) buffer. EDTA-treated wild-type (WT) or mutant enzymes (2 µM each) were incubated in a buffer containing 10 mM Tris–HCl (pH 7.4) and 5 mM 2-mercaptoethanol with different concentrations (0–10 mM) of Mg^2+^ or Ca^2+^ for 15 min at 25°C, and then fluorescence emission spectra were recorded. Control titrations were recorded in the presence of a monovalent cation Na^+^. All fluorescence emission spectra and fluorescence intensities from the titrations were corrected by subtraction of control spectra and control titrations.

### Electrophoretic mobility shift assay for DNA binding

Different concentrations of KpnI WT and its mutants were incubated with 5′ end-labeled double-stranded oligonucleotides (1 nM) containing canonical or non-canonical sites (20 bp) in binding buffer [10 mM Tris–HCl (pH 7.4) and 5 mM 2-mercaptoethanol] for 15 min on ice. The free DNA and the enzyme-bound complexes were resolved on 8% non-denaturing polyacrylamide gel in TBE buffer (89 mM Tris–HCl, 89 mM boric acid and 1 mM EDTA). Gels were visualized using a phosphorimager (Fujifilm, FLA5100).

### Surface plasmon resonance spectroscopy

DNA binding kinetics study was carried out by using surface plasmon resonance (SPR). A streptavidin matrix-coated sensor chip (BIAcore) was preconditioned with three consecutive injections of 1 M NaCl and further equilibrated with binding buffer [10 mM Tris–HCl (pH 7.4), 1 mM EDTA, 25 mM NaCl], at a flow rate of 10 µl/min. A 72mer 5′ biotinylated oligonucleotide (5′-ACT CTA GAG GAT CCC CGG TTG GAC TAA GTC CCC AGG CCC CTT AGC CAC CAC AAC GTG GGT ACC GAG CTC GAA-3′) was annealed with its complementary strand and was in turn immobilized onto the preconditioned streptavidin chip loaded into flow cell 2. Flow cell 1 contained an unmodified streptavidin chip as a reference surface. All binding studies were carried out in binding buffer using the BIAcore program Kinject. Increasing concentrations of the WT KpnI and its mutants were passed on to the chip at a flow rate of 50µl/min, and change in Response Units was monitored. The binding surface was regenerated each time by short pulses of 5 µl of 0.05% SDS. Data analysis was performed using BIA evaluation software version 3.1 (BIAcore AB) using 1:1 Langmuir interaction with mass transfer model. All SPR experiments were carried out at 25°C.

### DNA cleavage and kinetic analysis

DNA cleavage reactions were carried out by incubating WT or mutant KpnI proteins with pUC18 DNA (14 nM) (substrate carrying a single KpnI site) or 5′ end labeled oligonucleotide duplexes (10 nM) (Supplementary Table S1) in a reaction buffer containing 10 mM Tris–HCl (pH 8.0) and 2 mM MgCl_2_ or CaCl_2_ at 37°C for 1 h. The reactions were terminated using a stop dye containing 5% glycerol, 10 mM EDTA, 0.025% bromophenol blue and 0.025% xylene cyanol. The cleavage products of plasmid DNA and oligonucleotides were analyzed on 1% agarose and 12% urea polyacrylamide gel electrophoresis, respectively. Chemical modification reactions were carried out using diethylpyrocarbonate (DEPC), which modifies histidine residues to N-carbethoxy histidine. The WT enzyme and the mutants were pre-incubated with 5 mM of CaCl_2_ or MgCl_2_ for 15 min at 4°C in a 4-(2- hydroxy ethyl) piperazine ethane sulfonic acid (HEPES)-containing buffer before incubated with different concentrations of DEPC (25–100 μM) on ice for 2 min, and the treated enzymes were used for DNA cleavage in the presence of Mg^2+^. The Ca^2+^ chase reactions were carried out at a fixed concentration of Mg^2+^ (2 mM) and increasing concentrations of Ca^2+^ (0–10 mM). The cleavage products were analyzed on 1% agarose gel electrophoresis. Fidelity Index, defined as the ratio of the maximum enzyme amount showing no star activity to the minimum amount needed for complete digestion at the canonical recognition site, was determined as described (20). For the metal ion-dependence assays, the reactions were initiated by adding substrate DNA and transferring the reaction mixture to 37°C after incubating the enzyme with different concentrations of Mg^2+^ (0.01–10 mM) on ice for 5 min. The cleavage products of plasmid DNA and oligonucleotides were analyzed on 1% agarose gel and 12% urea PAGE, respectively.

Kinetic analysis was carried out at DNA concentrations of 7.5–250-fold molar excess over dimeric enzyme (1–10 nM) at 37°C in an assay buffer containing 10 mM Tris–HCl (pH 7.4), 25 mM NaCl, 5 mM 2-mercaptoethanol and 2 mM Mg^2+^. Aliquots were taken out at different time points for the determination of initial velocity. The reactions were terminated by adding an equal volume of stop dye containing 95% formamide, 10 mM EDTA, 1 mM NaOH, 0.025% xylene cyanol, 0.025% bromophenol blue. The kinetic parameters were determined by fitting the velocity with the substrate concentration using GraphPad Prism version 4. The turnover number (*k*_cat_*)* was calculated as the ratio of *V*_max_ to the dimeric enzyme concentration used (29).

### Molecular dynamics simulations

Four models of KpnI were created based on a homology model of KpnI complexed to its target DNA (4): KpnI WT with Ca^2+^ ions (WT-Ca^2+^), WT with Mg^2+^ ions (WT-Mg^2+^) and KpnI D148E mutant with Ca^2+^ ions (D148E-Ca^2+^) and Mg^2+^ ions (D148E-Mg^2+^), respectively. The metal ions have been placed at the same positions as observed in the crystal structure of I-PpoI complexed to DNA (PDB accession 1A73), and crystal water molecules observed in the active site of I-PpoI have been added. The mutant was modeled by replacing residue D148 with Glu *in silico*. We performed two control simulations of I-PpoI with Mg^2+^ ions and Ca^2+^ ions with the same settings as for KpnI.

The systems are each solvated in a cubic water box of size (x = y = z = 120 Å) using the explicit TIP3P water model (30). Forty-two Na^+^ counter ions were added to neutralize the system, and a further excess of Na^+^ and Cl^-^ ions was added so as to obtain a physiological concentration of 150 mM NaCl. The addition of the ions was carried out by random substitution of water oxygen atoms.

The simulations were run using the program NAMD (31) with the CHARMM27 force field (32). Simulations were performed using periodic boundary conditions and long-range electrostatic interactions were treated using the Particle Mesh Ewald method (33) on a 128 × 128 × 128 charge grid, with a non-bonded cutoff of 12 Å. Short range electrostatics and van der Waals interactions were truncated at 12 Å using a switch function starting at 10 Å. The solvated structures were minimized using 5000 steps of the steepest descent, followed by minimization with the conjugate gradient algorithm, with solute atoms harmonically reconstrained until an energy gradient of 0.01 kcal/(molÅ) was reached. The system was then gradually heated for 30 ps to 300 K with 1 K temperature steps with harmonic restraints on the solute atoms. Equilibration was performed in three stages with the numbers of particles, pressure (1 bar) and temperature (300 K) kept constant (*NpT* isothermal-isobaric ensemble) during a 75-ps window. In the first 25 ps, velocities were rescaled every 0.1 ps and in the second 25 ps, Langevin dynamics were used to maintain constant temperature. Pressure control was introduced in the third 25 ps and in the production run using the Nosé-Hoover Langevin piston (34) with a decay period of 500 fs. The harmonic restraints were gradually lifted [to 0.5, 0.25 and 0.05 kcal/(molÅ^2^)] in the three-equilibration stages. After equilibration, the NPT production runs were performed for 20 ns. The integration time step was 2 fs, and coordinates were saved with a sampling interval of 2 ps. All covalent bond lengths involving hydrogen were fixed using SHAKE algorithm. The two outer base pairs of the DNA were harmonically restrained to have the center of mass of the atoms forming Watson–Crick hydrogen bonds of around 3 Å. As the starting model only represents the inner (DNA binding) part of the protein, restraints were put on the Cα-atoms of residues A97-G141, D156-D163 and K179-N190 so as to mimic the stabilization by the missing residues. All other atoms were free to move. All molecular images were generated with Pymol [DeLano Scientific, Palo Alto, CA].

## RESULTS

### A conservative mutation of a metal ion-coordinating residue increases cleavage fidelity with Mg^2+^

**Figure 1.**
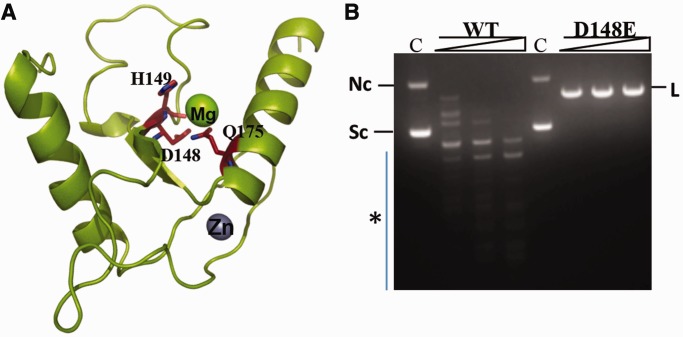
Mutant D148E exhibits high fidelity DNA cleavage. (**A**) HNH motif of KpnI. The KpnI monomer model is shown as a ribbon with helices as spirals and strands as arrows. The Mg^2+^ and Zn^2+^ ions are shown as spheres. The side chains of active site residues, D148, H149 and Q175, are shown as sticks and labeled. (**B**) Effect of D148E mutation of KpnI on DNA cleavage specificity. Plasmid DNA pUC18 (14 nM) was incubated with various concentrations (25, 50 and 100 nM) of WT or mutant enzyme for 1 h at 37°C in the presence of 5 mM Mg^2+^ and analyzed on 1% agarose gel. C indicates DNA cleavage reaction in the absence of enzyme. Nc, L and Sc indicate the positions of nicked circular, linear and supercoiled forms of the plasmid, respectively. The asterisk denotes the promiscuous DNA cleavage products.

The HNH active site of KpnI exhibits promiscuous cleavage activity in the presence of Mg^2+^ or high fidelity cleavage with Ca^2+^ (21). Sequence alignments, structural modeling and mutational analysis showed that H149 is the first histidine of the HNH motif in the enzyme that might function as a general base to activate a water molecule for the nucleophilic attack on the scissile phosphodiester bond (4,8). D148 and Q175 correspond to the metal-binding residues that coordinate the active site metal ion, which stabilizes the pentacovalent phospho-anion intermediate and assists in the protonation of the leaving group (Figure 1A). Previous studies with the enzyme revealed a correlation between ion size and promiscuous activity in the case of alkaline earth group of metal co-factors (19). In general, it was observed that metal ions of larger ionic radii induce high fidelity DNA cleavage. To address the role of metal ion coordination on the promiscuous activity, we mutated residue D148 to Glu such that the side chain is extended by a –CH_2_ group without altering its negative charge at neutral pH. When the DNA cleavage property of the mutant D148E was analyzed in the presence of 2 mM Mg^2+^, the enzyme showed no promiscuous activity even at high enzyme concentrations (Figure 1B). Fidelity index (FI) is defined as the ratio of the maximum amount of enzyme showing no star activity to the minimum amount of enzyme needed for complete digestion at the canonical recognition site (20). The FI of the mutant is ≥4000 in NEBuffer 4 [20 mM Tris acetate, 10 mM magnesium acetate, 50 mM potassium acetate, 1 mM DTT (pH 7.9)], compared with 4 for WT KpnI under the same reaction conditions (data not shown).

The mutation decreased the specific activity of the enzyme on the canonical substrate by ∼6-fold (Table 1). We carried out steady state kinetic studies using the canonical substrate to investigate the role of the mutation on the catalysis. The DNA cleavage reactions proceeded according to Michaelis–Menten kinetics, and the kinetic parameters were determined as described in ‘Materials and Methods’ section. The kinetic constants derived for the WT enzyme in the presence of Mg^2+^ were consistent with the previously reported values (29). The *K*_M_ value of the mutant decreased 2-fold from the WT value of 17.4 to 9.0 nM, whereas its *k*_cat_ value decreased 10-fold from 0.22 to 0.02, resulting in a decrease of *k*_cat_/*K*_M_ value by ∼4-fold (Table 2).

**Table 1.** Specific activity of the WT and mutants

Enzyme	Specific activity (units/mg)
WT	1.2 × 10^7^
D148E	1.8 × 10^6^
D16N	2.8 × 10^7^
D16N/D148E	1.2 × 10^7^

**Table 2.** Kinetic parameters for canonical DNA cleavage

Enzyme	K_M_ (nM)	*k*_cat_ (s^−1^)	*k*_cat_/K_M_ (s^−1 ^M^−1^)
			(×10^6^)
WT	17.4	0.22	12.64 ± 0.4
D16N	56.9	1.21	21.26 ± 0.2
D148E	9.0	0.02	3.22 ± 0.6
D16N/D148E	66.9	1.09	16.31 ± 0.6

### Mutation D148E abolishes cleavage activity with Ca^2+^

**Figure 2.**
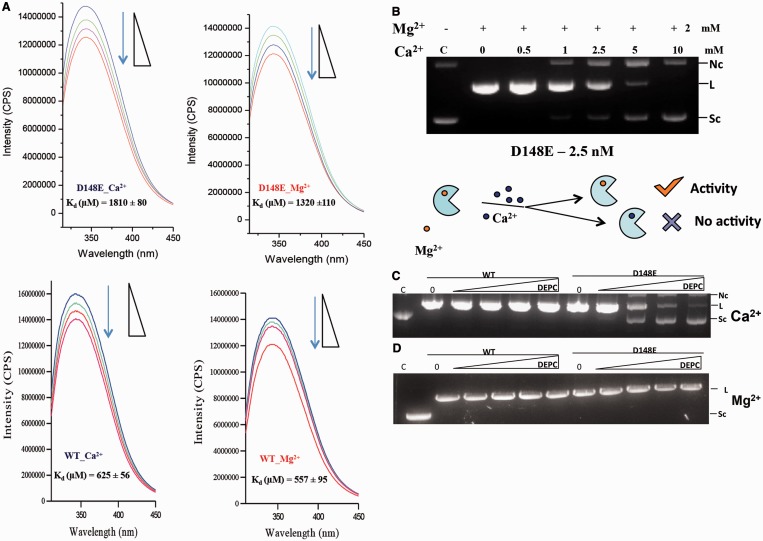
Ca^2+^ ion binds to KpnI D148E but does not support DNA cleavage. (**A**) Fluorescence emission spectra of WT and mutant D148E in the presence of Ca^2+^ and Mg^2+^ with increasing amounts of CaCl_2_ (Blue) or MgCl_2_ (Red) (0–10 mM). Representative spectra and the calculated K_d_ values are shown. (**B**) Metal ion competition assay. Reactions were carried out by assaying Mg^2+^-mediated DNA cleavage activity in the presence of titrating Ca^2+^ ions. Reactions contained 2.5 nM D148E and competition was performed by pre-incubation of the enzyme in a buffer containing 10 mM Tris–HCl (pH 7.4), on ice with the indicated metal ions. The reactions were initiated by addition of pUC18 DNA (14 nM) and incubated at 37°C for 1 h. Enhanced solvent accessibility of His149 in the presence of Ca^2+^. KpnI WT or mutant D148E enzymes were pre-incubated with 5 mM of (**C**) CaCl_2_ or (**D**) MgCl_2_ and treated with increasing concentrations (25–100 μM) of DEPC. Residual activity was assayed by cleavage of pUC18 DNA (14 nM) in the presence of 2 mM MgCl_2_.

Surprisingly, the mutant D148E did not show detectable DNA cleavage activity in the presence of 2 mM Ca^2+^ (Supplementary Figure S1). To determine whether the D148E mutation abrogated Ca^2+^ binding, Ca^2+^ or Mg^2+^ were titrated into mutant D148E, and intrinsic fluorescence of the protein was measured. Figure 2A shows that the mutant binds with similar affinity to both Mg^2+^ and Ca^2+^, but the affinity is lower compared with the WT, suggesting that the increase in the length of the side chain affects metal binding to some extent but does not abrogate Ca^2+^ binding. We have shown earlier that the change in the intrinsic fluorescence of the protein is mainly due to the metal ion binding to the HNH active site (25). Consistent to the comparable binding affinity of Ca^2+^ and Mg^2+^, Ca^2+^ could replace the Mg^2+^ bound at the HNH active site, resulting in the loss of activity (Figure 2B). These results suggest that Ca^2+^ ion binds to the active site of the D148E mutant as strongly as Mg^2+^ but forms a cleavage-incompetent enzyme-metal ion complex.

To investigate whether the Ca^2+^-bound D148E mutant enzyme has a different DNA binding property, electrophoretic mobility shift assays (EMSA) were carried out using 5′-end labeled 20 bp oligonucleotide duplexes containing a KpnI site. Ca^2+^ neither enhanced nor decreased the enzyme affinity for the canonical recognition sequence (Supplementary Figure S2), indicating that the metal ion could be affecting the next step, i.e. the DNA cleavage step.

We next tested whether the binding of Ca^2+^ affects the solvent exposure of H149 (36,37) through DEPC modification of histidine residues. In these experiments, we started with a low DEPC concentration to limit the modification to the active site histidine residue. When D148E KpnI was pre-incubated with Ca^2+^ and then treated with ≥50 μM of DEPC, the DNA cleavage activity with Mg^2+^ was inhibited (Figure 2C). Cleavage activity of WT KpnI was not affected at the same DEPC concentrations, suggesting that in mutant D148E, the general base H149 becomes more solvent exposed when Ca^2+^ is bound. However, in the presence of Mg^2+^, H149 was protected from DEPC inactivation in both WT and D148E (Figure 2D). The likely increase in solvent accessibility of H149 in the mutant suggests that the mutation induces a different Ca^2+^ coordination geometry not permissive to the formation of an active phosphodiester bond hydrolysis configuration.

### Molecular basis for high fidelity of KpnI D148E with Mg^2+^

**Figure 3.**
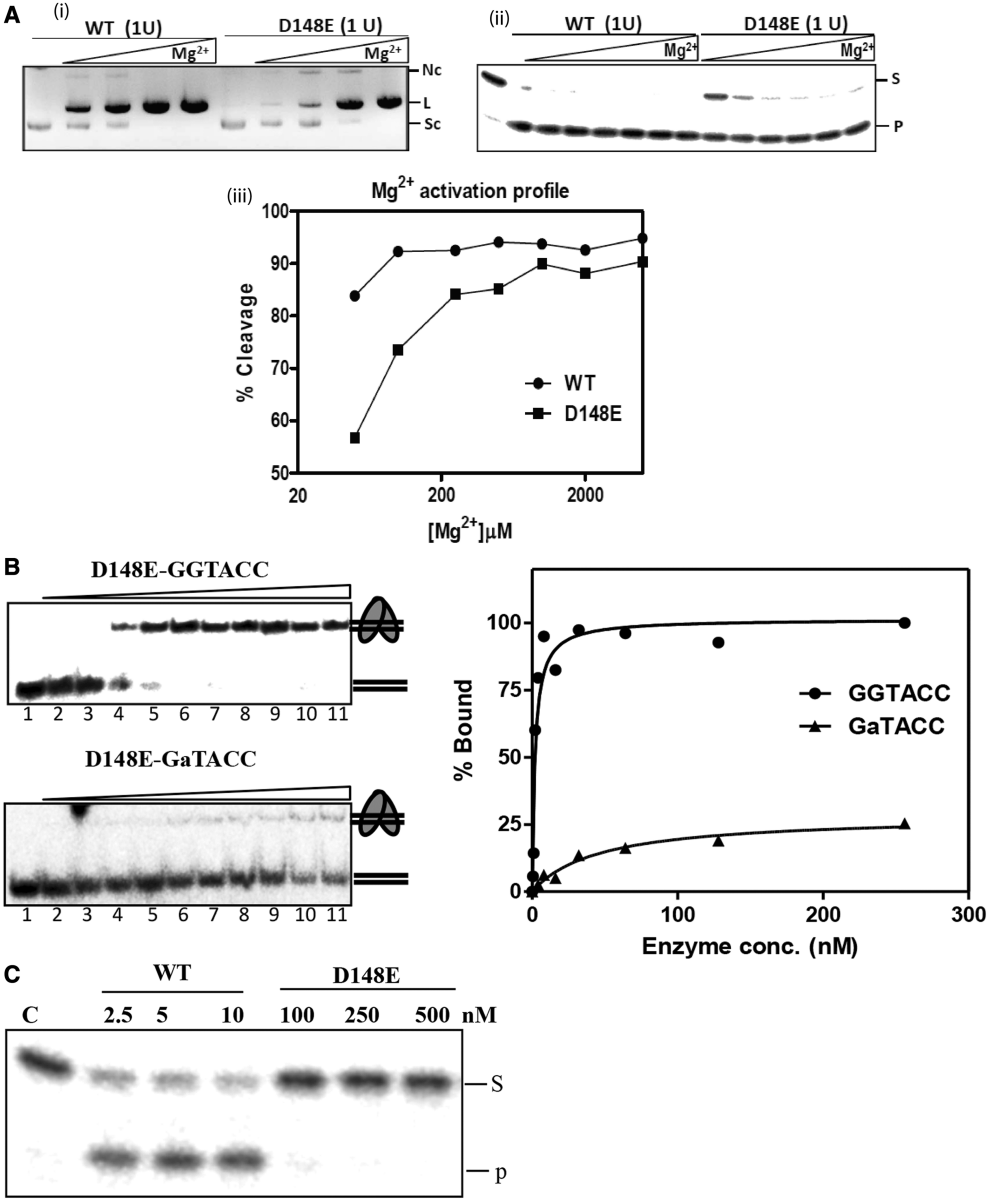
Molecular basis for the high fidelity of variant D148E. (**A**) Mg^2+^-dependent DNA cleavage of mutant D148E. Different concentrations of metal ions (0–10 mM) were incubated with 1 unit of WT or mutant D148E in a buffer containing 10 mM Tris–HCl (pH 7.4), on ice for 5 min. DNA cleavage reaction was initiated by the addition of (i) pUC18 DNA (14 nM) or (ii) end labeled canonical oligonucleotide (10 nM) and incubated at 37°C for 1 h. (iii) Graphical representation of the Mg^2+^ activation profile of WT and D148E variant on the oligonucleotide substrate. Mutant D148E requires a Mg^2+^ concentration of 200 μM for the 90% digestion of the substrate, compared with 50 μM required by WT, showing that the decrease in binding affinity toward Mg^2+^ (Figure 2A) directly affects the cleavage activity of KpnI. (**B**) Canonical versus non-canonical discrimination by mutant D148E. EMSA was carried to determine the binding affinity of mutant D148E to end-labeled canonical (GGTACC) and non-canonical (GaTACC) substrates over a range of enzyme concentrations between 0 and 256 nM. The intensity of the shifted DNA band is expressed as percentage bound. (**C**) Mutant D148E does not cleave star substrate at binding concentrations. End-labeled oligonucleotide duplex containing the 5′-GaTACC-3′ sequence (10 nM) was incubated with WT (2.5–10 nM) or mutant D148E (100–500 nM) at 37°C for 30 min in the presence of 2 mM Mg^2+^. The cleavage products were analyzed on 12% urea-PAGE. Lane C contains the substrate DNA without the enzyme. S and P represent the substrate and product, respectively.

Next, we investigated the molecular basis of the high fidelity achieved by the D148E mutant with Mg^2+^. To understand the effect of the increase in the side chain length at the 148 position on the Mg^2+^-mediated cleavage characteristics of the enzyme, the metal ion activation profile of the mutant D148E was compared with the WT enzyme. The proteins were pre-incubated with increasing concentrations of Mg^2+^, and the reactions were initiated by the addition of the substrate DNA. The WT enzyme exhibited complete cleavage of 14 nM of pUC18 DNA at Mg^2+^ concentrations as low as 50 µM. However, mutant D148E required ∼4-fold higher Mg^2+^ ion concentration for complete cleavage (Figure 3A).

Both the DNA binding and the cleavage steps can account for the fidelity of the REases. Increased discrimination of canonical from non-canonical sites and the relative rate of cleavage of the canonical and non-canonical sites determine the fidelity of an enzyme. We therefore carried out detailed kinetic studies for the binding and cleavage steps of WT and D148E KpnI. Equilibrium DNA-binding experiments using the canonical site in the absence of metal ions revealed that mutant D148E binds to the canonical site with an affinity of 2 nM, compared with very poor binding at a non-canonical site (GaTACC) (Figure 3B). This is in contrast to *K*_d_ values obtained for the WT enzyme, which binds to both canonical and non-canonical sites with high affinity of 9 and 13 nM, respectively (29). The differential binding of mutant D148E to the canonical and non-canonical sequences indicates a role for the residue D148 in target sequence discrimination. At higher enzyme concentrations, mutant D148E exhibited weak binding to the non-canonical DNA (Figure 3B), but the complex is resistant to cleavage (Figure 3C), suggesting that the mutation also suppresses promiscuous activity at the catalytic step.

### Molecular dynamics simulations show changes in active site geometry

**Figure 4.**
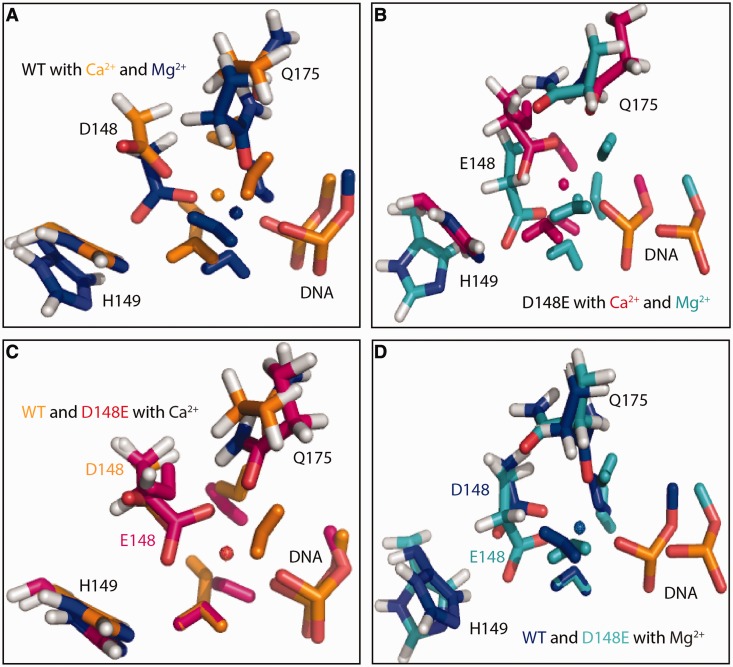
Molecular dynamics simulations for the interactions of KpnI WT and mutant D148E with Mg^2+^ and Ca^2+^. Representative snapshots of the catalytic pocket within the active sites of KpnI WT bound to Ca^2+^ and Mg^2+^ ion (**A**) and mutant D148E bound to Ca^2+^ and Mg^2+^. (**B**) A superimposition of WT and mutant D148E bound to Ca^2+^ or Mg^2+^ is shown in (**C**) and (**D**), respectively.

To understand how the mutation D148E alters the active site geometry, molecular dynamics simulations were performed for both Ca^2+^ and Mg^2+^ ions bound to WT (WT-Ca^2+^ and WT-Mg^2+^) and the mutant (D148E-Ca^2+^ and D148E-Mg^2+^), respectively. The simulations were performed based on the theoretical model of dimerized KpnI HNH active site bound to the target DNA (4). Representative snapshots of the catalytic pocket within the active sites of WT-Ca^2+^, WT-Mg^2+^, D148E-Ca^2+^and D148E-Mg^2+^are shown in Figure 4A and B, respectively. A superimposition of the WT and mutant D148E bound to Ca^2+^ or Mg^2+^ is shown in Figure 4C and D, respectively. The distance between the ions and key residues along the simulation time is given in Supplementary Figure S3.

Replacing Ca^2+^ with Mg^2+^ in the WT leads to small changes in the active site geometry and in particular to the coordination of the metal ion. From the WT-Ca^2+^ simulations, the metal ion is found to be coordinated by six atoms throughout the simulation time [coordination number (CN) = 6]: one oxygen atom of the phosphate group of the scissile phosphodiester bond of the DNA (Cyt13), one carboxyl oxygen atom of D148 and three water molecules throughout the simulation time (Supplementary Figure S3). A sixth coordination site is filled with Q175, occasionally replaced by a fourth water molecule. Simulations for the WT-Mg^2+^ complex indicate no change in CN, but Q175 coordinates the metal ion throughout the course of the simulation. In the control simulation of I-PpoI with Ca^2+^, the corresponding residue N119 was also observed to transiently leave the metal ion coordination sphere, whereas it remained close to Mg^2+^ in the entire simulation (Supplementary Figure S4). The distance between the metal ion and H149 (measured at the putative proton-acceptor nitrogen-atom) fluctuates ∼4.5 Å in both WT simulations, a distance that allows one water molecule in between. Our simulations showed that mutation D148E introduces a significant difference of the catalytic pocket between the two models with different metal ions and to the respective WT conformation (Figure 4C and D). The mutated residue Glu148 coordinates the Ca^2+^ ion with both carboxyl oxygen atoms throughout the simulation time in both subunits and coordinates the Mg^2+^ in one subunit. The extra coordination by the second Glu oxygen atom results in a CN of 7, whereas in the D148E-Mg^2+^ simulations a CN of 6 is maintained by having Q175 leaving the metal ion. In the D148E-Ca^2+^ simulations, the coordination by Q175 is lost and replaced by a fourth water molecule (Supplementary Figure S3). All other coordination bonds fluctuate ∼2 Å, indicating strong and consistent interactions between the DNA, Glu148 and the water molecules bound to the Ca^2+^ or Mg^2+^ ion.

### A suppressor mutation increases the specific activity of mutant D148E

Although the D148E mutation resulted in an enzyme with higher specificity, it had lower specific activity and *k*_cat_/*K*_M_ than the WT (Tables 1 and 2). We therefore set out to identify suppressor mutations that can reverse the reduced specific activity while retaining high fidelity. By targeted mutagenesis in the D148E background, eight mutants were found to have higher specific activity (data not shown). Surprisingly, DNA sequencing revealed that all the higher activity mutants had acquired an additional mutation D16N, suggesting that the higher specific activity was due to the D16N substitution but not the targeted mutations.

Site-directed mutagenesis was carried out to generate mutants D16N and D16N/D148E. In the case of D16N/D148E double mutant, the D16N suppressor mutation did not affect the other two properties of the D148E mutant, namely, the high substrate specificity in Mg^2+^ catalyzed reaction and the Ca^2+^-mediated inhibition of the cleavage activity (See Figure 1B and Supplementary Figure S1). The increase in the activity of the enzyme caused by mutation D16N could be the result of compensational interactions with the D148E mutation or enhanced activity independent of the D148E mutation or a combination of both. To delineate the mechanism, the DNA cleavage properties of the suppressor, D16N was evaluated in the context of WT and D148E mutation.

### Mutation D16N causes faster turnover

To better understand the role of the mutation D16N in improving specific activity, we carried out steady state kinetic analysis of mutants D16N, D148E, D16N/D148E and the WT enzyme using the canonical substrate (Table 2). The *K*_M_ values of the mutants D16N and D16N/D148E were 56.9 and 66.9 nM, respectively, compared with 17.4 and 9.0 nM of the WT and mutant D148E, respectively. The *k*_cat_ values of mutants D16N and D16N/D148E were 1.21 and 1.09 s^−^^1^, respectively, compared with 0.22 and 0.02 s^−^^1^ of the WT and mutant D148E, respectively. Compared with the WT enzyme, the *K*_M_ value of mutant D16N increased by ∼3-fold, whereas the *k*_cat_ value increased by 6-fold (Table 2). When compared with mutant D148E, the *K*_M_ value of mutant D16N/D148E increased by ∼7-fold, whereas the *k*_cat_ values increased by ∼37-fold, resulting in ∼5-fold improvement in *k*_cat/_*K*_M_ value. When the substrate concentration is higher than the *K*_M_ value, as in most commonly used cleavage conditions, the D16N/D148E enzyme cleaves the canonical site more efficiently than the D148E mutant owing to its higher *k*_cat_ value. Thus, introduction of D16N into the D148E background restored the *k*_cat_/*K*_M_ value in the double mutant to the WT level (Table 2).

**Figure 5.**
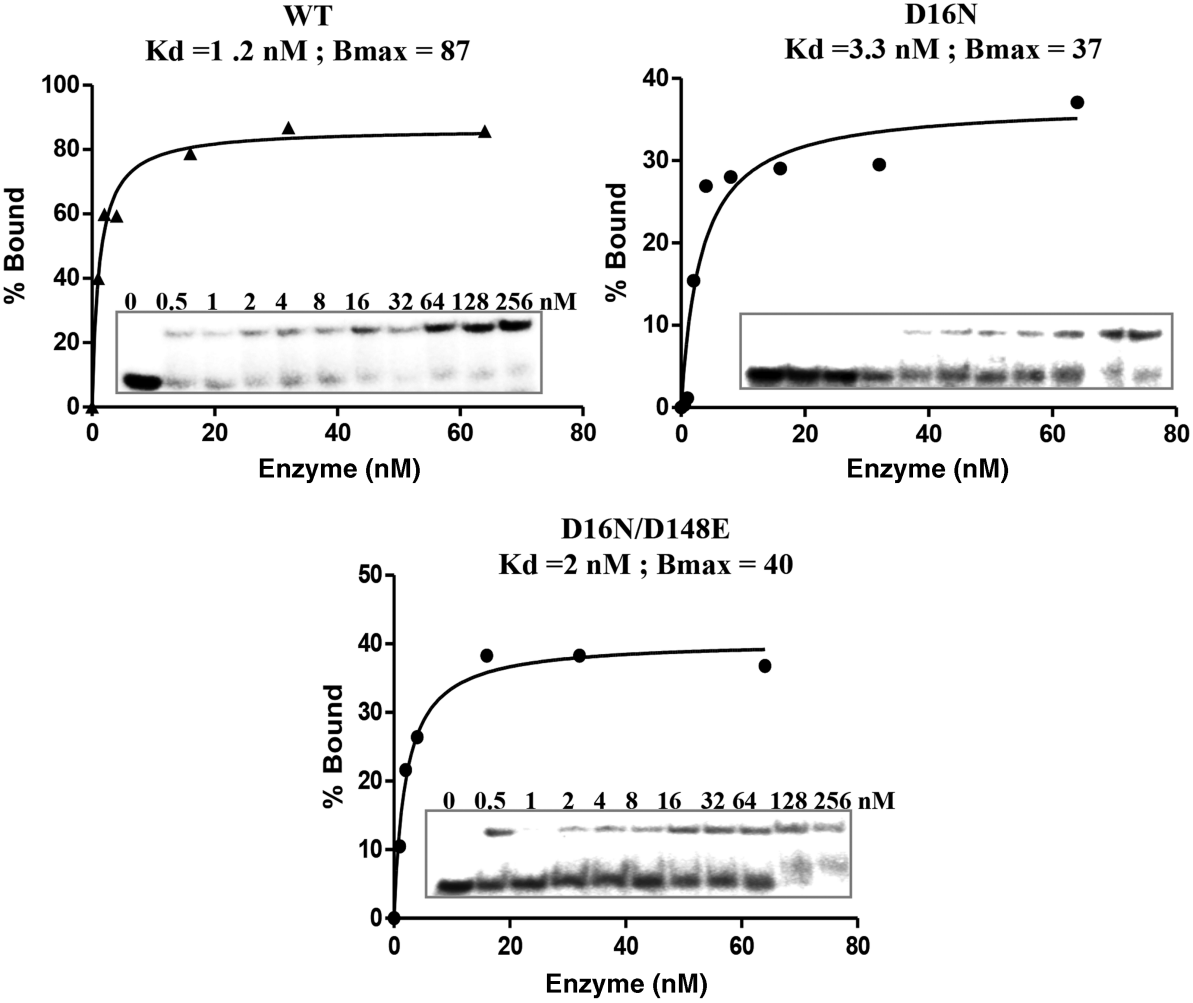
Suppressor mutant exhibits comparable DNA-binding affinity. EMSA analyses for the DNA-binding affinity of WT KpnI and mutants. Different concentrations of WT or KpnI mutants (0–256 nM) were incubated with 1 nM of end-labeled canonical (-GGTACC) oligonucleotide in binding buffer [20 mM Tris–HCl (pH 7.4), 25 mM NaCl and 5 mM 2-mercaptoethanol] on ice for 15 min before loading onto a 8% polyacrylamide gel. The intensity of the shifted DNA band is expressed as percentage bound.

**Figure 6.**
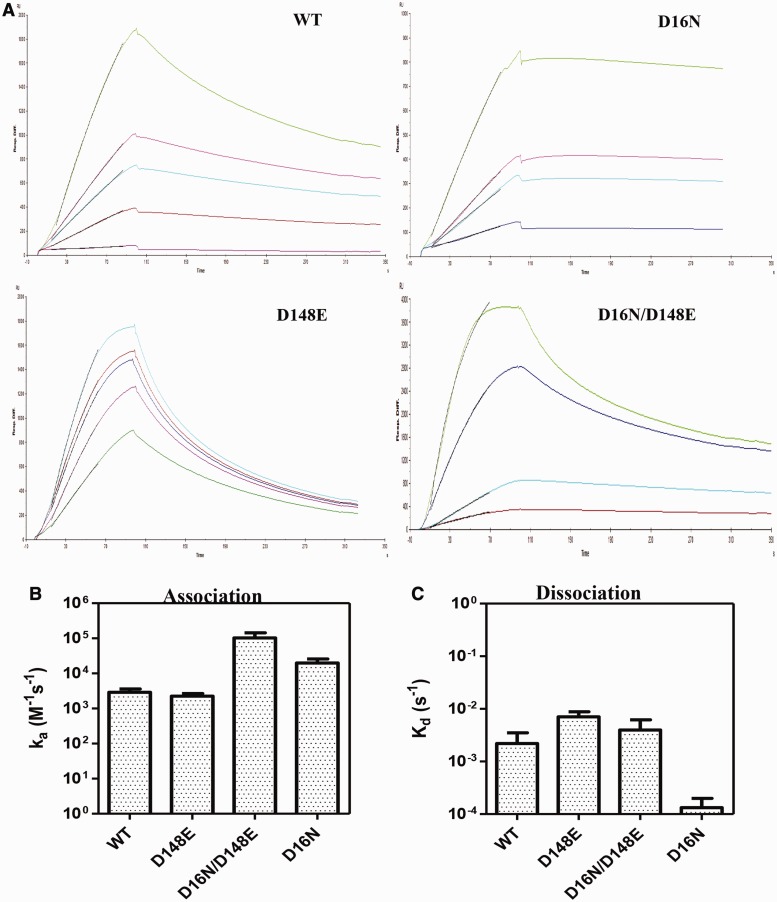
Binding kinetics to the canonical site. DNA-binding kinetics of WT KpnI and the mutants was assayed by SPR spectroscopy. Biotinylated oligonucleotide harboring the canonical (-GGTACC-) site was immobilized onto a streptavidin chip. The proteins were passed over the DNA with restricted flow rates to measure the association and dissociation rates, as described in ‘Materials and Methods’ section. (**A**) Sensorgrams of WT, D16N, D148E and D16N/D148E. Graphical representation of the association (**B**) and dissociation (**C**) rates of the proteins is shown.

To analyze the molecular basis for the increased specific activity, the DNA-binding property of the mutant proteins was assessed by EMSA. As shown in Figure 5, irrespective of their cleavage efficiency, all mutants bound to the canonical sequence with equilibrium binding affinity comparable with that of the WT enzyme. To further investigate the basis for the higher turnover number of the D16N mutation, the association and dissociation rates of WT, mutants D16N, D148E and D16N/D148E with the canonical recognition sequence were determined (Figure 6). At the association step, the WT enzyme and the mutant D148E exhibited comparable association rate (‘on’ rate). In contrast, the D16N and D16N/D148E mutants exhibited a 7- and 28-fold faster ‘on’ rates, respectively, compared with the WT enzyme, indicating that the D16N mutation increases the rate of association with the substrate DNA. At the dissociation step, when compared with WT enzyme, the D148E and D16N/D148E mutants exhibited 3.2- and 1.8-fold faster dissociation from the canonical DNA complex, respectively. In addition to a faster association rate, the D16N mutant exhibited a slower ‘off’ rate from the substrate, showing that the D16N mutation does not only speed up the formation of the KpnI-canonical DNA complex but also stabilizes the complex.

Lastly, limited proteolysis analysis of the WT, D16N, D148E and D16N/D148E was carried out to examine whether the enzymes have undergone major structural change. From Supplementary Figure S5, it is apparent that the trypsin cleavage patterns of the mutants were similar to that of WT, indicating that the increase in activity and sequence specificity is not due to major structural change to the protein.

## DISCUSSION

In this study, we show that a conservative substitution of Asp to Glu in the metal ion coordinating residue D148 of the catalytic/HNH motif of KpnI results in changes in two major aspects in its DNA cleavage activity—a drastic increase in cleavage fidelity with Mg^2+^ and elimination of cleavage activity with Ca^2+^. We show that the high fidelity cleavage was contributed by the enhanced discrimination of non-canonical sequences from the canonical at the binding step of the enzyme and at the catalytic step. Ca^2+^-mediated cleavage activity of the D148E mutant was abolished at the catalytic step. Molecular dynamics simulation data suggest that when Ca^2+^ is coordinated by Glu instead of Asp, it is more tightly bound (as indicated by the higher CN) and hence less likely to move toward the O3′ atom of the leaving group and facilitate its departure by compensating the negative charge. The D148E mutation decreases the turnover rate and *k*_cat_/*K*_M_ value of the enzyme, whereas the D16N mutation reverts the *k*_cat_/*K*_M_ value to the WT level through a higher turnover rate.

Generally, Ca^2+^ ions do not support the cleavage activity of PD-(D/E)-XK REases. However, it is well documented that members of the HNH superfamily of nucleases exhibit a wider metal ion preference and many of them can use Ca^2+^ ion as a cofactor (11,10,14–18), although the molecular basis of Ca^2+^-mediated DNA cleavage is not completely understood. In the case of KpnI, Ca^2+^ not only facilitates cleavage but also induces high cleavage fidelity (21). The binding of Ca^2+^ to KpnI mutant D148E, however, results in a catalytically inactive complex (Figure 2A and B and Supplementary Figure S1). Our results show that when a Ca^2+^ ion is bound to the D148E mutant, the side chain of H149 becomes more accessible to DEPC modification than the Ca^2+^-bound WT or the Mg2^+^-bound mutant D148E. We interpret the DEPC inactivation of mutant D148E by Ca^2+^ as mis-positioning of H149, the general base of the HNH active site (4), such that it cannot activate a nucleophilic water molecule for the cleavage reaction to occur. Alternatively, the ordered water molecule that acts as a nucleophile in the active site might be excluded due to the increased length of the acidic side chain of D148E when a Ca^2+^ ion is bound. Both of these mechanisms of inhibition have been proposed for other enzymes (35). For example, in the case of HincII and *E. coli* ribonuclease H1, it was observed that Ca^2+^ coordinates the general base required for catalysis (36,37). Comparison of the Mg^2+^ versus Ca^2+^ bound structure of the *E. coli* phosphoenolpyruvate carboxykinase revealed that the binding of a Ca^2+^ ion results in the exclusion of two water molecules at the active site (38). The increased accessibility of H149 to DEPC modification in mutant D148E favors our hypothesis that in D148E KpnI, H149 is mis-positioned such that it cannot act as a general base to activate the nucleophilic water molecule for cleavage to occur.

Based on kinetic, structural and/or biochemical studies, three possible mechanisms have been proposed to explain promiscuous DNA cleavage by REases. In the case of EcoRI, it has been shown that an overall increase in binding affinity of the enzyme to canonical, non-canonical or non-specific DNA sequence coupled with increased cleavage rate constants enhances the probability of cleavage at non-canonical sites (39). Studies with BamHI and its isochizomer, OkrAI have suggested that a structural element, the presence of a flexible C-terminal helix (as seen in BamHI) or its absence (in OkrAI), facilitates the formation of a non-canonical cleavage competent complex (40). Our previous studies have shown that binding of an additional Mg^2+^ to KpnI induces promiscuous activity (26). Mechanistically, a higher degree of recognition specificity can be the result of the enzyme’s discrimination of non-canonical sequences from the canonical one at the binding, cleavage or both steps. From the results presented here, it is evident that the high fidelity variant D148E exhibits reduced binding at non-canonical sites. The mutant D148E does bind to the substrate GaTACC at high enzyme concentrations but does not cut (Figure 3B and C), suggesting that the mutation also suppresses the catalytic step when a non-canonical substrate is bound. It is possible that the binding of the second Mg^2+^ ion required for inducing promiscuous activity (26,27) is hampered in the mutant. The change in the Mg^2+^ activation profile and the binding affinity toward Mg^2+^ and Ca^2+^ (Figure 3A) suggests that mutation D148E alters the metal ion coordination apparatus of the enzyme. Molecular dynamics simulations of the distances between the metal ions Ca^2+^ and Mg^2+^ and atoms of active site residues (Figure 4 and Supplementary Figure S3) agree with this proposition. In a typical one-metal ion mechanism, both the activation of the water molecule that becomes the nucleophile (by donating a proton to an acceptor residue such as a nearby histidine) and the compensation of the negative charge accumulating at the leaving group oxygen atom are achieved by the same metal ion. The leaving group can become protonated by a second metal-activated water molecule, or directly coordinated to the metal ion on dissociation of the P-O3′ bond. In either case, the metal ion must move somewhat from the attacking site to the departure site, requiring a subtle balance between metal ion mobility and coordination. Our molecular dynamics simulations show that the CN of the metal ion increases from 6 to 7 when Ca^2+^ is bound to mutant D148E. The higher CN may prevent ion movement, affecting the catalysis. Our simulation also indicated that mutation D148E introduces significant changes of the active site pocket and the mode of Mg^2+^ ion coordination with respective to the WT conformation (Figure 4D and Supplementary Figure S3). How these changes in the D148E-Mg^2+^ complex are related to the reduced specific activity and better discrimination against non-canonical substrates? More detailed simulations and biochemical experiments will be needed to investigate this issue.

Although D148E eliminates cleavage activity with Ca^2+^ and promiscuous activity with Mg^2+^ and retains similar affinity to both the metal ions, we have shown previously that mutating D148 to Gly eliminated KpnI’s cleavage activity with Mg^2+^ by preventing the binding of the metal ion to the active site. The latter mutant showed lower cleavage activity with non-canonical sites with Mn^2+^ and lower ds cleavage activity toward canonical substrate, leading to the accumulation of nicked intermediates (25). These properties of D148 mutants underscore the important role of the residue in the plasticity of the metal ion cofactor selection and the enzyme’s promiscuous activity, and suggest a correlation between the bound metal ion cofactor and promiscuous activity in KpnI.

It is noteworthy that other residues or motifs outside of the HNH motif also play a role in the cleavage fidelity and Ca^2+^-mediated activity of KpnI. First, mutation of residue D163 to Ile resulted in the loss of Ca^2+^-mediated activity and high cleavage fidelity with Mg^2+^ (26,27). Second, mutation in a separate motif ExDxD (E132, D134 and D136), which is involved in Ca^2+^ coordination reduced the Ca^2+^-mediated cleavage activity of canonical substrate and Mg^2+^-mediated promiscuous activity (27). Although it is known that Ca^2+^ coordinates with a larger number of amino acid residues compared with Mg^2+^ due to its larger atomic radius, structural information is required for a better insight into the distinct features of different metal ion-mediated activity in KpnI.

The higher *k*_cat_ values of mutants D16N and D16N/D148E (compared with WT and mutant D148E, respectively) indicate that the D16N mutation increases the turnover of the enzyme. Without an atomic structure or a reliable structural model of the N-terminus of KpnI, we are not able to suggest a molecular mechanism of how the D16N mutation increases the turnover of the enzyme on its canonical substrate. The higher on rate and lower off rate of the mutant D16N toward the cognate substrate suggests that the D16N mutation alters the DNA-binding interactions. Recent studies with acylphosphatases have shown that removal of a salt-bridge near the active site decreases the activation barrier of the catalytic reaction (41).

To conclude, the present study describes a mutation that results in altered metal ion coordination and elimination of promiscuous activity for restriction enzyme KpnI. Together with our previous studies, we propose that the plasticity of the KpnI active site for metal ion cofactor is associated with its promiscuous activity. Substitution(s) of residues that are involved in co-factor coordination in the enzymes could be a general strategy to improve the substrate specificity of HNH REases and possibly other HNH nucleases. Demonstration that spontaneous mutations distal to the active site of the enzyme influence the rate of catalysis opens up further avenues for enzyme engineering. Approaches discussed here for the generation of variant enzymes would thus form the basis for future studies. The tremendous effect of a single point mutation on the abolition of non-canonical DNA cleavage but retention of high sequence specificity emphasizes the role of promiscuous activity in divergence of function while maintaining native interactions.

## SUPPLEMENTARY DATA

Supplementary Data are available at NAR Online.

## FUNDING

Work carried out at the Nagaraja lab in India Institute of Science was supported by the J.C. Bose fellowship grant from Department of Science and Technology, Government of India. Work carried out at New England Biolabs was supported by NEB. Funding for open access charge: New England Biolabs will pay for the publication charges.

*Conflict of interest statement*. Several of the authors (S.H.C., Z.Z. and S.Y.X.) are employees of New England Biolabs; the KpnI enzymes are commercial products sold by that company.

## Supplementary Material

Supplementary Data
