# Supplementary material for: Increasing cleavage specificity and activity of restriction endonuclease KpnI
Source: Nucleic Acids Res. 2013 Aug 19;41(21):9812–24. doi: 10.1093/nar/gkt734 (PMC3834813; doi:10.1093/nar/gkt734)
Supplement: Supplementary Data [file supp_41_21_9812__index.html]

Increasing cleavage specificity and activity of restriction endonuclease KpnI — Increasing cleavage specificity and activity of restriction endonuclease KpnI — Supplementary Data 

# Increasing cleavage specificity and activity of restriction endonuclease KpnI

## Supplementary Data

files

**Files in this Data Supplement:**

- Supplementary Data - pdf file
