# Supplementary material for: Increasing cleavage specificity and activity of restriction endonuclease KpnI
Source: Nucleic Acids Res. 2013 Aug 19;41(21):9812–24. doi: 10.1093/nar/gkt734 (PMC3834813; doi:10.1093/nar/gkt734)
Supplement: Supplementary Data [file supp_gkt734_nar-01369-f-2013-File013.pdf]

Figure S1

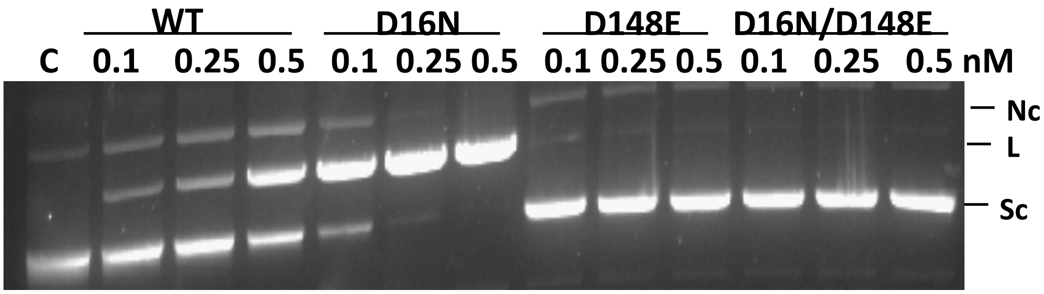

Figure S1. DNA cleavage properties of KpnI and its mutants in the presence of Ca^{2+}. pUC18 DNA (14 nM) cleavage was carried out with different concentrations of WT and mutants as indicated in the presence of 2 mM Ca^{2+}. The reactions were incubated at 37 °C for 30 min and analyzed on 1% agarose gel. Nc, L, and Sc indicate the positions of nicked circular, linear, and supercoiled forms of the plasmid, respectively.

Figure S2

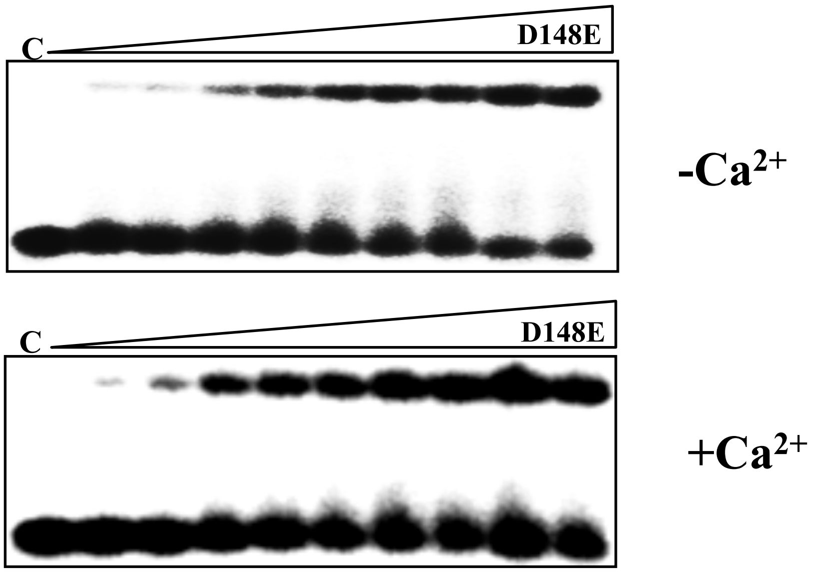

Figure S2. Effect of Ca^{2+} on the DNA binding ability of mutant D148E. Different concentrations of KpnI were incubated with 1 nM of end labeled canonical oligonucleotide duplexes in the absence or presence of inhibitory concentrations (2 mM) of Ca^{2+} in binding buffer (20 mM Tris-HCl, pH 7.4, 25 mM NaCl, and 5 mM 2-mercaptoethanol) on ice for 15 min before analyzed on a native polyacrylamide gels.

Figure S3

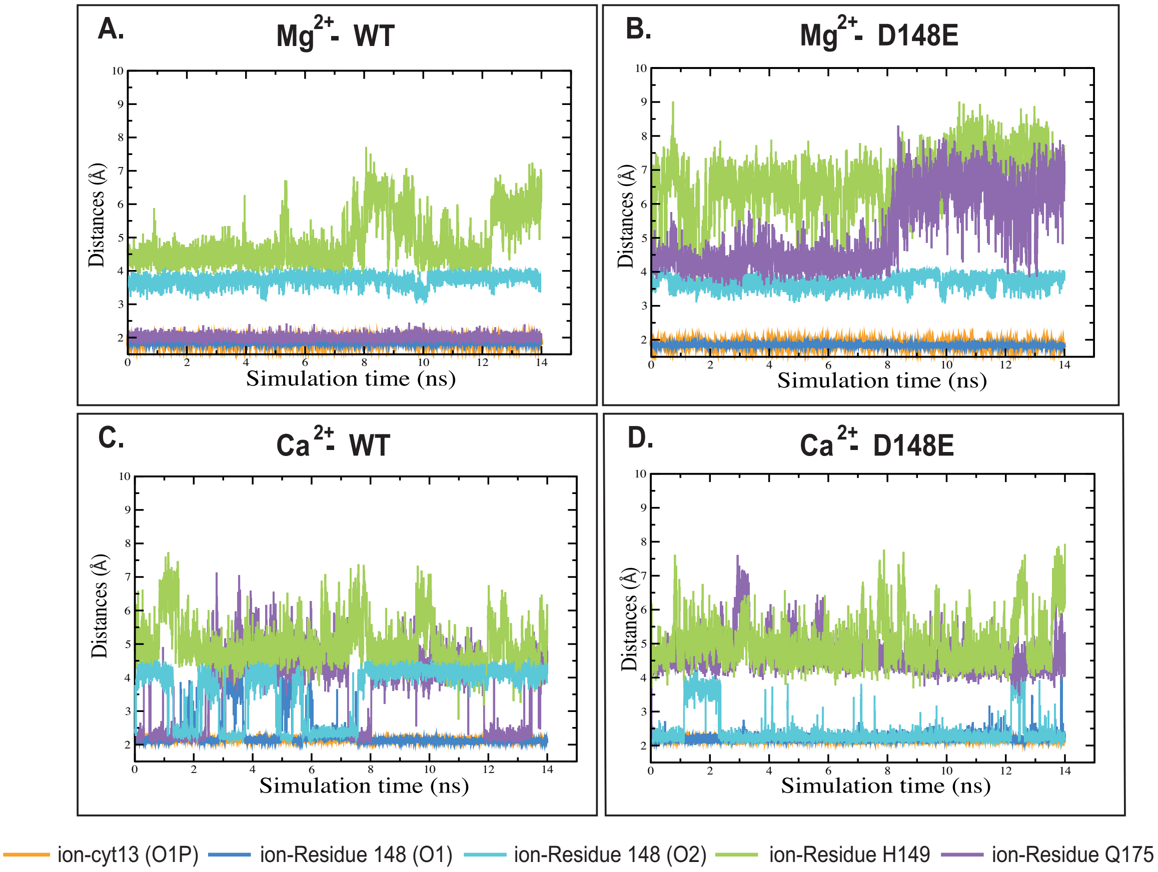

Figure S3. Molecular dynamics simulations of the distance between the metal ions and oxygen atoms of active site residues. The distance between the metal ion, oxygen atom of the phosphate group of the scissile phosphodiester bond Cyt13 (orange), Asp/Glu148 O1 (dark blue), Asp/Glu148 O2 (light blue), His149 NE (green) and Q175 OE1 (purple) over 14 ns simulation time are plotted for Mg^{2+} with WT (A) and mutant D148E (B), and for Ca^{2+} with WT (C) and mutant D148E (D), respectively.

Figure S4

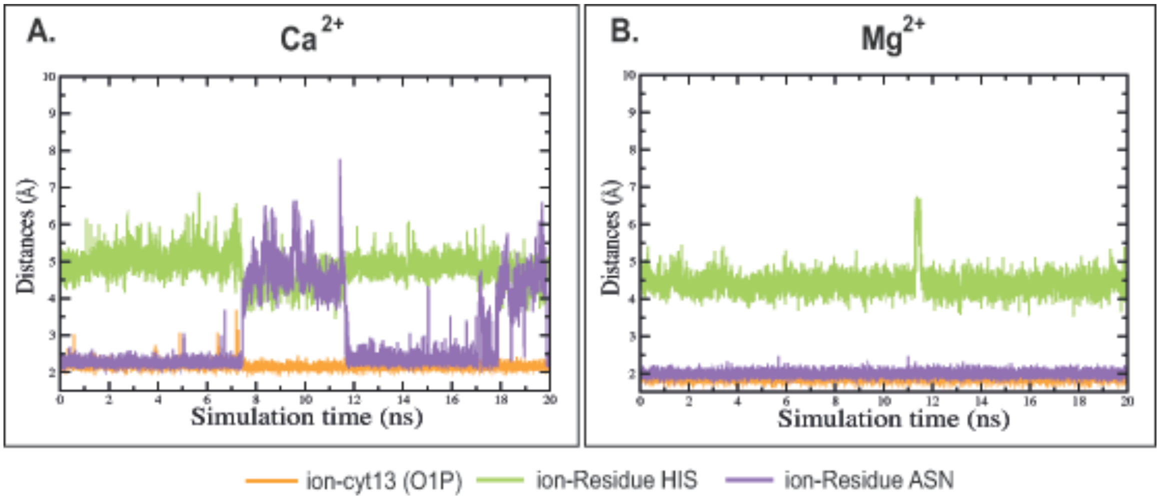

Figure S4. Distance between the metal ions and oxygen atoms of active site residues in the MD simulations of PpoI. The distance between the metal ion, oxygen atom of the phosphate group of the scissile phosphodiester bond Cyt13 (orange), His (green) and Asn (purple) of I-PpoI over 20 ns simulation time are plotted for Ca^{2+} (A) and for Mg^{2+} (B), respectively.

Figure S5

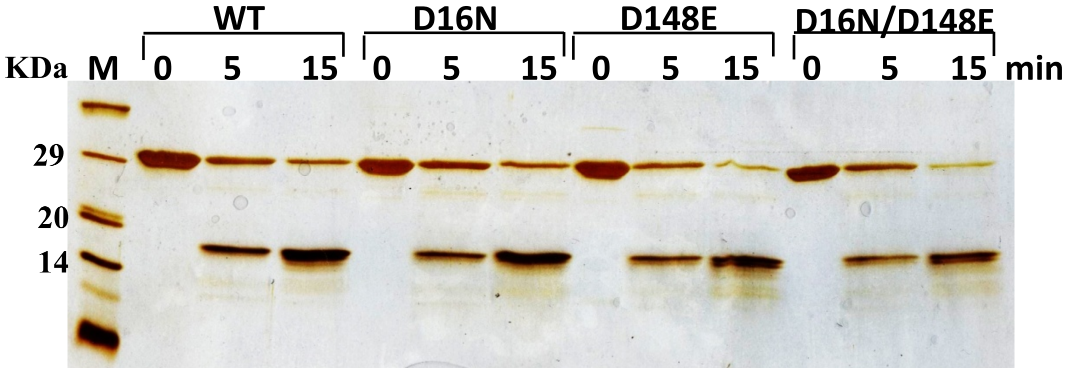

Figure S5. Trypsin digestion profiles of KpnI and its mutants. Proteolytic digestions of different KpnI and its mutants (1 mg/ml) were carried out in 50 mM Tris-HCl buffer, pH 8.5, and at 37°C using 2% (w/w) trypsin. 5 μl samples of the reaction mixture were taken after various time intervals and proteolysis was stopped with SDS-PAGE loading buffer containing PMSF. Samples were analyzed by 15% SDS-PAGE and visualized by silver staining.

Supplementary Table S1

Oligonucleotide duplex used for EMSA and DNA cleavage	
Canonical	5 ' – ATTGCGT**GGTACC**CGCTCTT – 3 '<br>3 ' – TAACGCA**CCATGG**GCGAGAA – 5'
Non-canonical	5 ' – ATTGCGT**GaTACC**CGCTCTT – 3 '<br>3 ' – TAACGT**CtATGG**GGCGAGAA – 5'
